# Functional Testing of ETV6 Variants: Is the Evaluation of Their Intracellular Localization Sufficient in Assessing Pathogenicity?

**DOI:** 10.3390/genes16020112

**Published:** 2025-01-21

**Authors:** Daniele Ammeti, Tonia De Simone, Stefania Zampieri, Cristina Bon, Melania Eva Zanchetta, Roberta Bottega, Michela Faleschini, Anna Savoia

**Affiliations:** 1 Institute for Maternal and Child Health, IRCCS Burlo Garofolo, 34137 Trieste, Italy; daniele.ammeti@burlo.trieste.it (D.A.); stefania.zampieri@burlo.trieste.it (S.Z.); cristina.bon@burlo.trieste.it (C.B.); melaniaeva.zanchetta@burlo.trieste.it (M.E.Z.); roberta.bottega@burlo.trieste.it (R.B.); 2 Department of Engineering for Innovation Medicine, University of Verona, 37134 Verona, Italy; tonia.desimone@univr.it

**Keywords:** *ETV6*, thrombocytopenia, functional studies

## Abstract

Background/Objectives: ETV6-related thrombocytopenia (ETV6-RT) is a rare autosomal dominant disorder characterized by mild thrombocytopenia since birth and an increased predisposition to hematologic malignancies. ETV6 functions as a transcriptional repressor, and its pathogenic variants, predominantly within the ETS domain, disrupt nuclear localization and transcriptional activity. In individuals with congenital thrombocytopenia, we identified two missense variants: c.1110A>G, p.Ile370Met, a novel variant, and c.1133G>A, p.Arg378Gln, a known variant with conflicting pathogenicity interpretations, for which functional characterization is necessary to provide an accurate molecular diagnosis. Methods: In silico bioinformatic tools and structural modeling were used to predict the impact of the variants. Functional assays included a dual-luciferase reporter assay to measure transcriptional activity and immunofluorescence/immunoblotting to assess intracellular localization in transfected HEK293T and HeLa cells. Results: Bioinformatic predictions and structural analyses suggested that the two variants might play a role in altering the folding or function of the ETS domain. Functional analysis revealed that p.Ile370Met abolished the ETV6 transcriptional repression activity, confirming its pathogenicity. p.Arg378Gln had no effect on the reporter gene levels, and, as expected, it localized in the nucleus. Interestingly, unlike the known mutations, which fail to enter the nucleus, p.Ile370Met retained partial nuclear localization. Conclusions: Since we described the first ETV6 mutation localized in the ETS domain that causes a loss of transcriptional activity, although it maintains the ability to enter the nucleus, we suggest that both transcriptional activity and intracellular localization assays are important for the accurate classification of *ETV6* variants by functional studies.

## 1. Introduction

ETV6-related thrombocytopenia (ETV6-RT) is an autosomal dominant form of inherited thrombocytopenia (IT) caused by mutations in *ETV6* (*ETS* variant 6) gene [1], encoding a master hematopoietic transcriptional repressor structured in three functional domains: the N-terminal pointed domain (PNT), the regulatory domain (or central regulatory domain CRD), and C-terminal DNA-binding domain (ETS). ETV6 was initially identified as a tumor suppressor well known for its role in chromosomal translocations associated to childhood leukemia, but it also plays an important role in hematopoiesis. Although ETV6 binds the promoter of many important genes involved in erythropoiesis and platelet production, its involvement in human megakaryopoiesis remains poorly described [2,3].

The ETV6-RT phenotype is characterized by a mild form of thrombocytopenia without alteration in platelet function. Although no syndromic clinical features are present, it is associated with an increased risk of developing hematological neoplasms, particularly B-cell acute lymphoblastic leukemia [4,5,6,7].

Since its first description [8,9], novel variants of *ETV6*, often of uncertain significance (VUS), and mostly located in the ETS domain, emerged from individuals with suspected inherited thrombocytopenia. Therefore, it is necessary to couple the mutational screening to specific functional analysis to define their pathogenic role.

To date, specific functional analyses were mainly addressed to assay the impact of the aminoacidic change in the residual transcriptional activity and the intracellular location of the mutant transcription factor [5,8,9,10,11,12]. Indeed, the ETV6 mutants lose their repressive activity and retain protein in the cytoplasmic compartment.

In this study, we tested the pathogenicity of the c.1110A>G (p.Ile370Met) and c.1133G>A (p.Arg378Gln) variants by analyzing their cellular localization and repressive activity [13]. Although we did not observe any effect of p.Arg378Gln, we found that, while p.Ile370Met fails to repress its targets as the other described mutations, it still maintains the ability to enter the nucleus. These observations highlight the importance of evaluating both the transcriptional activity and subcellular localization of ETV6 variants to assess their pathogenicity.

## 2. Materials and Methods

### 2.1. Genetic Analysis

The search for mutations was conducted by targeted sequencing using the MiSeq platform (Illumina). The sequencing panel included 28 genes correlated with hereditary plateletopenias, as previously described [13]. All reported variants were confirmed by Sanger Sequencing.

### 2.2. Bioinformatic Tools

Bioinformatic analysis was performed using a Variant Effect Predictor (VEP, v113.0) at https://grch37.ensembl.org/info/docs/tools/vep/index.html (accessed on 5 December 2024) and evaluating the following software: PolyPhen-2 (Polymorphism Phenotyping, v2.2.2), SIFT (Sorting Intolerant From Tolerant, v5.2.2) and CADD (Combined Annotation Dependent Depletion, v1.7).

The crystal structure of ETS domain of ETV6 bound to a specific DNA sequence (PDB ID 4MHG) was analyzed.

### 2.3. Plasmids

The wild-type (WT) full-length *ETV6* cDNA with a 5′Myc Tag sequence was subcloned from a lentiviral vector kindly provided by W. Kahr and inserted into a pcDNA3.1 (+) expression vector (Invitrogen, Carlsbad, CA, USA). *ETV6* mutants (p.Ile370Met and p.Arg378Gln) were generated by PCR site-directed mutagenesis using primers designed with Quick Change Primer Design (Agilent) software (https://www.agilent.com.cn/store/primerDesignProgram.jsp, accessed on 5 December 2024).

The target *MMP3* promoter region was cloned upstream the *Photinus pyralis* luciferase gene in the PGL4 basic vector (Promega, Madison, WI, USA). Reporter Vector expressing *Renilla reniformis* luciferase under the control of the constitutive promoter CMV (pRL-CMV) was bought from Promega.

### 2.4. Cells Cultures

Human Embryonic Kidney 293T (HEK293T) cells and HeLa were grown using Dulbecco’s Modified Eagle’s Medium (DMEM) supplemented with 10% Fetal Bovine Serum (FBS) and 100 units/mL Penicillin/Streptomycin. Medium and supplements were provided by Euroclone S.p.A. (Pero, Italy). All cells were kept in culture in humified incubators at 37 °C and 5% of CO_2_.

### 2.5. Transfection Methods

HEK293T cells were plated onto 6cm dishes (plating cells density 9 × 10^5^ cells/well) and after 24 h were transfected using Polyethylenimine (PEI) in a ratio of 3 μL/1 μg of DNA. After 12 h of incubation, cells were washed with PBS and transferred to fresh medium.

Lipofection was performed to transfect HeLa cells with expression vector using Lipofectamine 3000 (Invitrogen) following protocol provided by the company.

### 2.6. Immunoblotting

HEK293T cell lysates were obtained using M-PER™ Extraction Reagents (Thermo Fisher Scientific, Waltham, MA, USA), and nuclear and cytoplasmatic protein fraction were obtained using NE-PER™ Nuclear and Cytoplasmic Extraction Reagents (Thermo Fisher Scientific) following the protocol provided by the company.

Then, 4× Laemmli sample buffer (BioRad, Hercules, CA, USA) was added to lysates and heated to 95 °C for 10 min. Then, we performed an SDS-PAGE using Mini-PROTEAN-TGX precast-gel (BioRad). After separation, proteins were transferred onto a nitrocellulose membrane (Trans Blot Turbo Transfer Pack, BioRad) with Trans Blot Turbo Transfer System (BioRad), and then the membrane was blocked with non-fat milk (1× PBS, 0.2% Tween20 and 5% non-fat milk). After blocking, the membrane was incubated overnight at 4 °C with primary antibody (anti-c-Myc (9E10); anti-HSP90 (F-8) or anti-SP1 (PEP2), Santa Cruz Biotechnology, Dallas, TX, USA) then was incubated with secondary antibody conjugated with horseradish peroxidase (HRP) (anti-mouse or anti rabbit, Santa Cruz Biotechnology). The membrane was then washed and finally incubated with HRP substrate (Clarity or Clarity Max, Biorad). The signal was detected with ChemiDoc Imaging System (BioRad).

### 2.7. Dual-Luciferase Reporter Assay

The assay for the transactivation activity was performed using the dual-luciferase reporter assay system according to the manufacturer’s instructions (Promega) as described before [10,14]. Briefly, HEK293T cells were plated on a 24 well plate at a density of 5 × 10^4^. Then, after 24 h, cells were transfected with 240 ng of each ETV6 expression vector, together with 72 ng of PGL4 and 24 ng of pRL-CMV. After 48 h, cells were lysed with 100 μL of Passive Lysis Buffer (PLB) 1X provided by the company. The assay for the transactivation activity was performed using the dual-luciferase reporter assay system according to the manufacturer’s instructions (Promega). The results were expressed as ratio of *P. pyralis* to *R. reniformis* luciferase (LucF/LucR) and graphically displayed by histograms, representing the mean value of at least three independent experiments with standard deviations. Statistical analysis (unpaired *t*-test) was performed using GraphPad Prism software (v8.0.2).

### 2.8. Immunofluorescence Assay

HeLa cells were plated onto 1.7 cm^2^ chamber culture slides (Corning, Corning, NY, USA) (cells density 5 × 10^4^ cells/well) and, after 24 h, were transfected with 1 μg of cDNA expression vector. Then, after 48 h, cells were fixed cells with 4% paraformaldehyde for 20 min, and cells were permeabilized with 0.1% Triton X-100 and treated for at least 30 min with a blocking buffer composed by 3% FBS in PBS. Cells were then incubated with primary antibody for 2 h and secondary antibodies for 1 h at 37 °C in a wet chamber. Nuclei were stained with Hoechst staining solution incubating 5 min. Chamber culture slides were finally covered with liquid mountant (ProLong, Thermo Fisher Scientific) and a cover slip.

To detect Myc-tagged ETV6 mouse, monoclonal antibody 9E10 (anti-c-Myc) (Santa Cruz Biotechnology) was used. As secondary antibodies, Alexa Fluor 488 (A10034) (ThermoFisher Scientific) was used.

To obtain images, a Zeiss Axioplan 2 epifluorescence imaging microscope was used. The images were acquired using a 40X Plan-NEOFLUAR objective and a Zeiss Axiocam 506 color was used. Images processed using Zeiss ZEN v3.1 (blue edition). Brightness and contrast regulation using Adobe Photoshop, v21.0.0.

## 3. Results

### 3.1. Bioinformatic Prediction and Structural Analysis

In thrombocytopenic patients referred to our institute, analyzed using a next-generation sequencing (NGS) approach for molecular diagnostic purposes, we identified two *ETV6* missense variants: c.1110A>G (p.Ile370Met), a novel aminoacid substitution, and c.1133G>A (p.Arg378Gln), which is reported in ClinVar (https://www.ncbi.nlm.nih.gov/clinvar/, accessed on 5 December 2024) with a conflicting interpretation of pathogenicity. Accordingly, the bioinformatics software tool for the clinical interpretation of genetic variants based on ACMG guideline (InterVar, https://wintervar.wglab.org/, accessed on 30 December 2024) classifies both variants as variants of uncertain significance (VUS) (Table 1).

**Figure 1:**
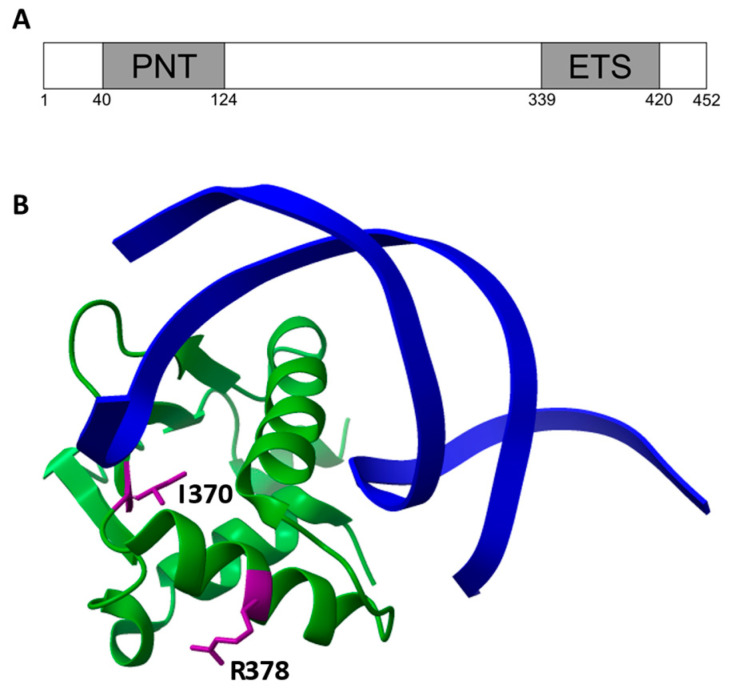
(**A**) Schematic representation of ETV6 domains. Amino acid number of the domain boundaries are reported below. (**B**) Structural analysis of the aminoacidic changes on ETS domain. Structural analysis of ETS bound to a specific DNA sequence (PDB ID 4MHG). The DNA cognate is reported in blue, the ETS domain is reported in green, and the residue involved in the aminoacidic change is highlighted in purple.

In both cases, they affect the ETS domain of ETV6 (Figure 1A), and in silico prediction tools do not exclude their pathogenic role (Table 1), with SIFT supporting the deleterious effect of only p.Ile370Met, while PolyPhen2 and CADD scores support the damaging effects of both variants.

Structural analysis of the ETS domain of ETV6 bound to a specific target sequence (PDB ID 4MHG) revealed that neither of the two mutated amino acids are involved in electrostatic interactions with the DNA cognate (Figure 1B). The substitution of isoleucine 370, residing in the hydrophobic core of the ETS, with a methionine does not introduce changes in overall hydrophobicity; however, the longer side chain with a higher steric hindrance, together with the presence of the sulfur atom, could destabilize its folding. The arginine 378, laying on an α-helix trait well exposed to the solvent, should not participate in interaction with other chains. However, the aminoacidic change cause a loss of a positive charged which could alter the stability or the function of the domain.

Although bioinformatic and structural analysis suggests that the variants could have some role in the pathogenicity, only functional studies would validate the predictions of different tools.

### 3.2. Mutant ETV6 Transcriptional Activity

To assess the pathogenicity of *ETV6* variants on the transcriptional activity of ETV6, we set up a dual-luciferase assay [8,10], cloning the *MMP3* promoter, a validated target of ETV6 [15] upstream to the *P. pyralis* luciferase cDNA.

**Figure 2:**
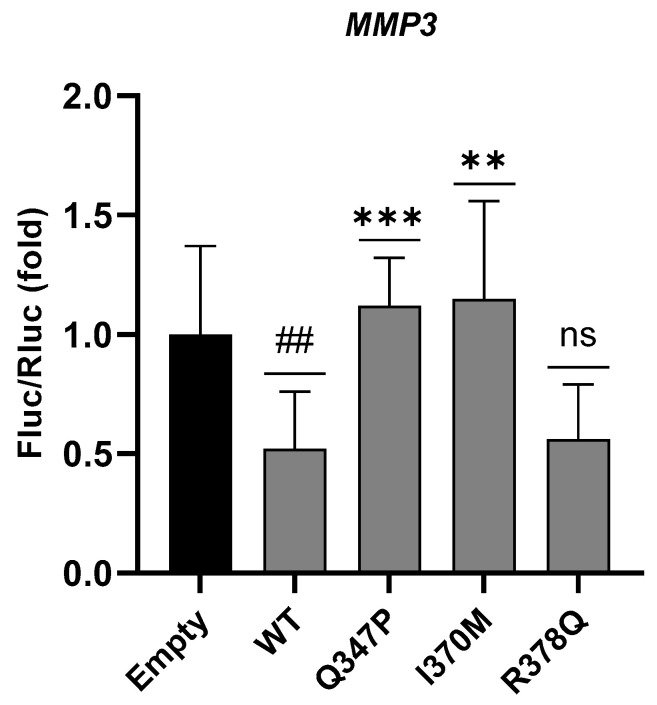
Effect of ETV6 mutation on the repressive activity on *MMP3* promoter. Bars represents the *P. pyralis*/*R. reniformis* luciferase emission ratio in HEK293T cells with firefly luciferase expression controlled by the *MMP3* promoter. Ratios are normalized to emission ratio of the empty vector (black bar). Mutations are reported as follows: p.Gln347Pro: Q347P; p.Ile370Met: I370M; and p.Arg378Gnl: R378Q. Error bars indicate the standard deviation from three independent experiments. Significance levels are represented as ## = *p* < 0.01 compared to the empty vector, and ns = *p* > 0.05, ** = *p* < 0.01, *** = *p* < 0.001 compared to WT-ETV6 overexpression, using Student’s *t*-test.

While HEK293T cells transiently overexpressing the wild-type (WT) protein showed a reduction in luciferase activity of 54 ± 24% compared to the empty control, luciferase activity levels after transfection with p.Ile370Met ETV6 were significantly higher (115 ± 41%) and comparable to that observed with the known p.Gln347Pro mutant protein used as control [10]. Instead, p.Arg378Gln ETV6 did not show any significant alteration in luciferase activity compared to that observed transfecting the WT protein (Figure 2). These results support the pathogenicity of the p.Ile370Met variant, which abolishes the transcriptional activity of ETV6, while p.Arg378Gln does not have any effect on the repression of the *MMP3* promoter.

### 3.3. ETV6 Mutant Intracellular Localization

To further confirm the results obtained before, we tested the intracellular localization of ETV6, which is expected to be cytoplasmic for the ETV6 mutations [10].

**Figure 3:**
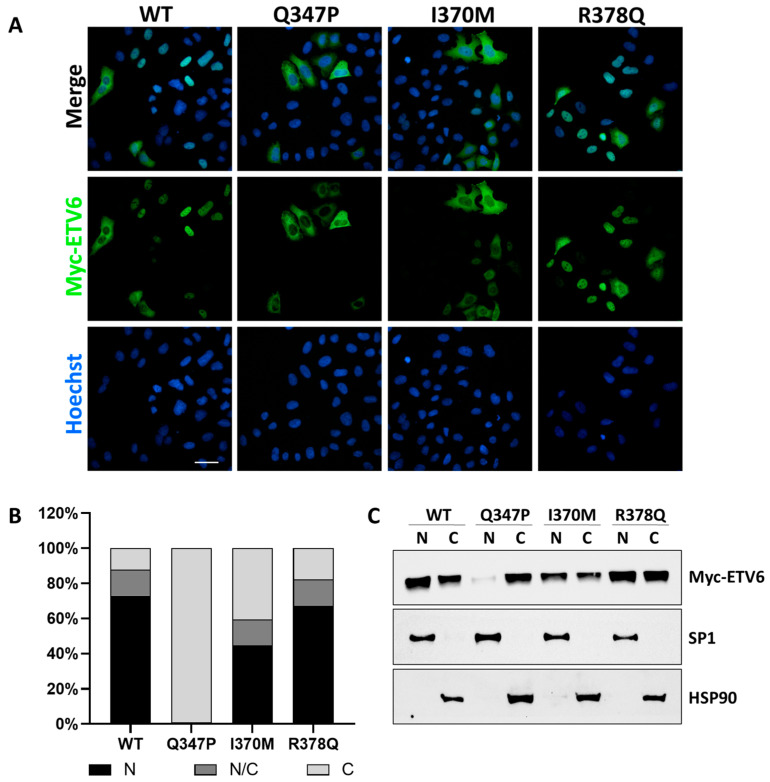
Functional analyses of the impact of ETV6 mutation on its intracellular localization. (**A**) Immunofluorescence analysis in HeLa cells transfected with Myc-tagged ETV6 constructs cloned into the pcDNA3.1 expression vector. Myc-ETV6 is shown in green, and nuclei are stained in blue (Hoechst staining). Images shown are representative of three independent experiments. Scale bar = 100 µm. (**B**) Quantification of ETV6 subcellular localization in cells. The histogram represents the proportion of cells with nuclear (N, black), cytoplasmic (C, light gray), or mixed nuclear/cytoplasmic (N/C, gray) staining. Data are based on results from three independent experiments counting at least 100 cells. (**C**) Western blot analysis of nuclear and cytoplasmic fractions from HEK293T cells 48 h post-transfection with wild type or mutant Myc-tagged ETV6. HSP90 and SP1 were used as markers for cytoplasmic and nuclear fractions, respectively. Mutations are reported as follows: p.Gln347Pro: Q347P; p.Ile370Met: I370M; and p.Arg378Gnl: R378Q.

First, we transiently overexpressed either the WT or mutant forms of ETV6 in HeLa cells to assess the ETV6 intracellular distribution through immunostaining analysis (Figure 3A,B). As expected, WT protein displayed a nuclear staining in 72.9% of the cells whereas the control mutant p.Gln347Pro protein showed almost total cytoplasmic localization (98.9%).

Regarding the novel variants tested, while p.Arg378Gln staining was comparable to that observed in WT-transfected cells, the p.Ile370Met maintained a nuclear staining in 44.6% of the cells, higher than expected for an ETV6 mutation.

These data were confirmed by immunoblotting the nuclear and cytoplasmic fractions in HEK293T cells after the overexpression of WT or mutant ETV6 (Figure 3C).

## 4. Discussion

Since its first description in 2015 [8,9], many novel mutations of *ETV6* gene were identified in patients with thrombocytopenia and/or acute leukemia, mostly clustered in the gene region encoding the ETS domain [4,5].

From that moment on, the pathogenic mechanism has been deeply investigated, and although many aspects remain unclear, it has been established that the effect of mutations is due to the delocalization of ETV6 in the cytoplasm, with the consequent loss of its ability to bind DNA and perform its transcriptional functions. This effect is amplified by the fact that ETV6 acts as a dimer, so mutations not only fail to exert their functions but also sequester part of the wild-type protein into a non-functional heterodimer, resulting in a negative dominant effect [5,8,9,10,16].

In this context, we analyzed two variants of ETV6, p.Ile370Met and p.Arg378Gln, both localized in the ETS domain, classified as VUS by ACMG criteria and predicted to have effects on the function of the transcription factor. Moreover, whereas no substitutions of 370 residue have been reported, the p.Arg378Gln is enlisted in ClinVar as a variant of conflicting interpretation, though the same amino acid replaced by a proline (R378P) has been assessed to be damaging [5]. However, since the R378 residue lays on an α-helix trait, the presence of the proline probably disrupts the structure of the ETS domain, while the aminoacidic change from arginine to glutamine is tolerated.

The functional analysis performed with the same assays used to assess the pathogenicity of other *ETV6* mutations [5,8,9,10,11] allowed us to conclude that p.Arg378Gln probably has no effect on ETV6 function and, therefore, can be regarded as a benign variant of *ETV6*, at least for the aspects investigated in this study. Instead, p.Ile370Met, affecting the ETV6 transcriptional activity at the same level as other known mutations, compatible with a dominant-negative effect [10], was classified as pathogenic. However, it only partially alters its nuclear localization. To date, the delocalization from the nuclear compartment to the cytoplasm is a well-established pathogenic mechanism in ETV6-RT. The nuclear exclusion is mediated either by the inability of ETV6 to reach the nucleus due to mutations in the ETS domain [10] or the formation of a novel nuclear export signal in the case of the most recurrent mutation outside the ETS domain, p.P214L [17]. Consistently, the immunostaining of ETV6 in patients’ platelets—generated from the fragmentation of the cytoplasm of megakaryocytes carrying mutant *ETV6*—has been proposed as a functional validation assay for the pathogenicity of *ETV6* variants [18].

Although all previously described mutants occurring in the ETS domain have been observed to not translocate into the nucleus, this new mutation raises new concerns. In particular, it suggests that the only localization of the transcription factor without testing its transcriptional activity could lead to the erroneous classification of variants.

In conclusion, we identified a novel ETV6 mutation, p.Ile370Met, and clarified that p.Arg378Gln does not impact the protein function, at least in terms of transcriptional activity. Moreover, we assessed that p.Ile370Met is the first pathogenic ETV6 mutation detected at significant levels in the nucleus, suggesting that the pathogenic effect of variants should be determined by evaluating different aspects of the protein function.

## Figures and Tables

**Table 1:** In silico prediction score and interpretation of pathogenicity of *ETV6* variants. Variants are annotated on reference sequence NM_001987.5. VUS: variant of uncertain significance.

Variant	Protein	dbSNP	SIFT	PolyPhen2	CADD PHRED	Clinvar	InterVar
1110A>G	p.Ile370Met	nr	0 (deleterious)	0.993 (probably damaging)	24.8	nr	VUS
c.1133G>A	p.Arg378Gln	rs146280653	0.08 (tolerated)	0.865 (possibly damaging)	31	Uncertain significance (2); Likely benign (2))	VUS

## Data Availability

All data relevant to the study are included in the manuscript and are available upon request to the corresponding authors.
